# Kernel-based Gaussian process for anomaly detection in sparse gamma-ray data

**DOI:** 10.1371/journal.pone.0228048

**Published:** 2020-01-23

**Authors:** Gregory R. Romanchek, Zheng Liu, Shiva Abbaszadeh

**Affiliations:** 1 Department of Nuclear, Plasma, and Radiological Engineering, Grainger College of Engineering, University of Illinois at Urbana-Champaign, Urbana, Illinois, United States of America; 2 Department of Electrical and Computer Engineering, Jack Baskin School of Engineering, University of California Santa Cruz, Santa Cruz, California, United States of America; University of Queensland, AUSTRALIA

## Abstract

In radioactive source surveying protocols, a number of task-inherent features degrade the quality of collected gamma ray spectra, including: limited dwell times, a fluctuating background, a large distance to the source, weak source activity, and the low sensitivity of mobile detectors. Thus, collected gamma ray spectra are expected to be sparse and noise dominated. For extremely sparse spectra, direct background subtraction is infeasible and many background estimation techniques do not apply. In this paper, we present a statistical algorithm for source estimation and anomaly detection under such conditions. We employ a fixed-hyperparameter Gaussian processes regression methodology with a linear innovation sequence scheme in order to quickly update an ongoing source distribution estimate with no prior training required. We have evaluated the effectiveness of this approach for anomaly detection using background spectra collected with a Kromek D3S and simulated source spectrum and hyperparameters defined by detector characteristics and information derived from collected spectra. We attained an area under the ROC curve of 0.902 for identifying sparse source peaks within a sparse gamma ray spectrum and achieved a true positive rate of 93% when selecting the optimum thresholding value derived from the ROC curve.

## Introduction

Advanced source surveying protocols are crucial tools in the various national security scenarios for which the presence and location of radioactive material must be quickly acquired. However, several factors inherent to source surveying degrade or otherwise limit the quality of information available. Focusing on surveying with a single mobile detector (as opposed to detector networks), there is a distinction between two prominent surveying strategies: static protocols and dynamic protocols.

Static protocols, such as the uniform search method [1], are deployed identically in every surveying scenario and do not involve a decision-making process. Dynamic protocols, on the other hand, adaptively adjust pathing based on a predetermined model and acquired data in order to locate the source in as few steps or in as little time as possible. These provide faster localization time than the static counterparts [2, 3]. For both protocol types, dwell times are often very short (on the order of seconds) [2–4]. Combined with a large distance to the source and the low sensitivity of portable detectors, collected spectra will be background dominated and contain very few counts. In these cases, dynamic protocols can falter due to lack of relevant information (source counts) compared to the presence of noise (background counts). Because of this, dynamic protocols often default to static protocols like a uniform search method or random walk until greater source information is present [2, 3]. Thus, the benefits of dynamic protocols cannot be realized until they leave this static period of pathing. So, minimizing the static portion of dynamic protocols is critical.

The quality of source information can be improved by either increasing source presence or reducing background presence in the spectrum. Reducing background presence is feasible in source surveying scenarios but comes with a set of problems. No matter how sensitive the detector is, naturally occurring radioactive material (NORM) is an omnipresent and unpredictable (on short time scales) source of radiative counts. Gammas belonging to the NORMs of ^40^K, daughters of ^238^U, ^232^Th, ^235^U, and more dominate the background energy spectrum from 0 to 3 MeV [5–7]. Background subtraction techniques can either estimate the net background counts in a gross tally or predict the distribution of the full background spectrum for analysis. In exceedingly sparse data (less than 100 counts/s), however, the total background count can fluctuate greatly from one sampling to the next; and the counts in a given channel will also have high relative variance. Low source-to-background counts rates may also hide relevant photopeaks such that background subtraction techniques remove these source peaks as well.

While there are computational methods for background estimation, many fail to meet the established criteria for source localization problems as they require either defined peak regions [8], the tuning of parameters [9], or rely on fully-developed spectra [10]. This is unsatisfactory for source-search problems as the specific Special Nuclear Material (SNM) present may not be known, and thus its photopeak region cannot be selected. Secondly, manually tuning or training parameters requires knowledge of the given system/algorithm, predictable data, and time, none of which may be present in emergency situations. Finally, given extremely sparse spectra, many of the provided techniques of background removal simply do not function as they rely upon features within high-count spectra.

*Alamoniotis et al*. [11], however, developed a kernel-based Gaussian process (KBGP) for background estimation under sparse conditions and with no prior information. Their quantification of “sparse,” however, (~435 background counts/s achieved with a 3 in × 3 in NaI detector) is more than four times that achieved with handheld detectors such as the Kromek D3S [12] which are capable of being hoisted with drones (quadcopters). Thus, the Kromek D3S, which has already been deployed in mobile sensor networks [13–15] provides a better representation of the background count rate (~50 counts/s) for the mobile detectors likely to be used in source surveying scenarios. This definition of “sparse” is a necessary consequence of mobile source surveying, and so a method for analyzing this level of sparse spectra is needed.

In terms of increasing source presence in the collected spectra, the unique feature of source localization protocols acquiring an independent spectrum sample at each new testing location can help. Since a number of gamma spectra are collected over the total surveying period you can view these collections as a sequence of noisy samples of the true source spectrum distribution. For each individual spectra collected, source data may be insignificant; but using all the samplings collectively can improve the confidence of estimated source presence. Combining this with sparse data background removal provides a statistical framework for estimating source presence in surveying protocols.

In this paper, we present a statistical algorithm for estimating source presence under the sparse conditions inherent to source surveying protocols. Background presence is first reduced in collected spectra by using a KBGP structure presented in [11] but further optimized for the sparse data at hand. Each newly acquired noise-reduced collection is then used to update an ongoing source distribution estimate via a Linear Innovation Sequences (LIS) scheme for a KBGP estimation. For this algorithm, we assume no prior information on background or source, and the source distribution is estimated purely based on the readings acquired while surveying. KBGPs were selected for both noise removal and source distribution prediction as they require no previous training and are computationally inexpensive, allowing the algorithm to be executed by on-board equipment in real-time.

Alternative machine learning algorithms exists which have the potential to estimate source spectra from collected spectra through both regression and categorical approaches, such as increasingly popular neural networks. With the conditions set out previously, however, such techniques are not well suited for this application. Namely, any machine learning methodology with substantial training requirements would hinder the generalizability of the algorithm. With substantial variation in background activity and composition, diverse combinations of source type and activity, and biases inherent to the specific detector type used, too many variables come into play to reasonably incorporate sufficient training examples to learn all cases accurately. In addition to the breadth of encounterable spectra, time dependencies need to be taken into account, adding a dimension of complexity to the training data required. The KBGP presented allows us to bypass the training phase and view generated source spectra as a statistical inference rather than a learned prediction on a mission-to-mission basis.

## Materials and methods

The following sections first provide an overview of Gaussian processes, kernel functions, hyperparameters, and LIS, followed by a description of the algorithmic methodologies. For the remainder of this paper, let: 1) the survey period denote the total time spent gathering gamma spectra while seeking the source, 2) the dwell time be the collection time for each individual gamma spectrum, and 3) a collection refer to one spectrum acquired during one dwell time. For example, collection *n* refers to the *n*^*th*^ gamma spectrum acquired while surveying and always has a sampling period of one dwell time.

### Kernel-based Gaussian processes

The goal of regression problems is to estimate the function *f*, which is defined by *y* = *f*(*x*) and represents the relationship between an input *x* and output *y*. Estimating this relationship is typically accomplished by constraining a class of test functions and then optimizing the function parameters by using training data to produce the smallest error. One downside of this technique is that it only optimizes function parameters and not the function selection itself, leaving the user to manually pick which functions to test. Gaussian process (GP) regressions, on the other hand, consider all possible functions simultaneously and compute a prior probability over this set such that higher probabilities dictate a greater likelihood that the function represents the data. This approach is more flexible, as it does not constrain the estimation to certain classes of functions [16, 17].

To understand GP regression, first, consider the general regression set-up:

$$y=\sum_{m=1}^{M}w_{m}\phi_{m}\left(x\right)$$ (1)

Here, *y* is the scalar output representing the observations, ***x*** = {*x*_1_,…,*x*_*N*_} is an *N*-dimesnional input representing the features, *ϕ*_*m*_ are the basis functions representing a mapping from feature space to a transformed space defining ***ϕ*** = {*ϕ*_1_(***x***),…,*ϕ*_*M*_(***x***)}, *w*_*m*_ are the associated scalar weights defining ***w*** = {*w*_1_,…,*w*_*M*_}, and *M* is the number of basis functions considered. As an example, the linear regression *y* = *mx*+*b* can be derived from Eq (1) by considering ***x*** = {*x*} (single feature) and ***ϕ*** = {*ϕ*_1_(*x*), *ϕ*_2_(*x*)}, where *ϕ*_1_(***x***) = *mx* and *ϕ*_2_(***x***) = *b*. Typically, we are given *D* training samples with: ***x***^(1)^,…,***x***^(*D*)^ where ***x***^(*d*)^ = {*x*_1_,…,*x*_*N*_}, and corresponding observations ***y*** = {*y*_1_,…,*y*_*D*_}. Thus, training sample 1 is {***x***^(1)^,*y*_1_}. In the context of our problem, we consider the single input feature of energy channel such that ***x***^(*d*)^ = {*x*_*d*_} and the corresponding observation *y*_*d*_ to be the counts in this channel. One full spectrum consists of observations {*x*_*d*_,*y*_*d*_} for *d* = {1,…,*D*} where *D* is the number of detector channels. Our goal is to first remove the background counts in channel *x*_*d*_ and then predict the source presence. Eq (1) can be represented in vector form as:

y=Φw (2)

where **Φ** is the *D*×*M* design matrix whose elements are Φ_(*d*,*m*)_ = *ϕ*_*m*_(***x***_*d*_) where *m* = {1,…,*M*} and *d* = {1,…,*D*}. GPs allow us to avoid defining the basis functions, and so the final estimate will be independent of *M*.

From here, we move toward deriving the GP estimator by casting the regression problem into a Bayesian formalism. A normal prior distribution over the weight vector takes the form:

$$P(w)=N(0,\sigma_{w}^{2}I)$$ (3)

The mean of which is zero, and each weight has uniform variance $\sigma_{w}^{2}$. This implies each weight is uncorrelated and that their distribution is governed by the hyperparameter $\sigma_{w}^{2}$. We assume this prior distribution for the weights due to the lack of prior information [2, 3]. Since Eq (2) is now defined as a linear combination of jointly Gaussian variables, ***y*** itself is Gaussian. Thus, its expectation value and variance are [16]:

E[y]=E[Φw]=ΦE[w]=0 (4)

$$cov(y)=E[(y-E[y]){\left(y-E[y]\right)}^{T}]=E[yy^{T}]=\Phi E[ww^{T}]\Phi^{T}=\sigma_{w}^{2}\Phi \Phi^{T}=K$$ (5)

where **K** is the *D*×*D* Gram Matrix with elements:

$$K_{\left(i,j\right)}=k(x_{i},x_{j})=\sigma_{w}^{2}\phi {\left(x_{i}\right)}^{T}\phi (x_{j})$$ (6)

where *i*,*j* = {1,…,*D*} and *k*(***x*,*x***′) is the kernel function with scalar output. Kernel representation condenses the inner product in feature space without needing to explicitly define the feature mapping ***ϕ*** itself. This inner product, the kernel function, is the covariance of ***x*** and ***x***′ in the transformed space [16]. The prior over our output vector ***y*** then follows the distribution:

P(y)=N(0,K) (7)

In random processes, however, there is often assumed to be some noise within the observations of the target values such that:

$$t_{d}=y_{d}+\epsilon_{d}$$ (8)

where ***ϵ*** represents the noise in observations ***y***, and each element *ϵ*_*d*_ are assumed to be uncorrelated with each other and normally distributed with mean zero and variance $\sigma_{d}^{2}$. Physically, for an observation of counts *y* in channel *x*, *y* is not necessarily the true count expected in *x* but rather a sampling of a random variable that can take a range of values. The prior over ***t*** = {*t*_1_,…,*t*_*D*_} is subsequently [16]:

$$P(t)=N(0,K+\sigma_{d}^{2}I)$$ (9)

Since the goal of GP is regression, we want to predict new values in ***t*** given features ***x***. Suppose you are given the output vector ***t*** = {*t*_1_,…,*t*_*D*_} and corresponding input vector ***x*** = {***x***_1_,…,***x***_*D*_}.We would like to estimate point *t*_*D*+1_ for a new input ***x***_*D*+1_; in other words, we want to compute the probability distribution for *t*_*D*+1_, *P*(*t*_*D*+1_|***t***), given training set {***x***,***t***} and testing point ***x***_*D*+1_. From this probability distribution, we can acquire an estimated mean and variance for *t*_*D*+1_. By conditional probability:

$$P(t_{D+1}|t)=\frac{P({t,t}_{D+1})}{P(t)}$$ (10)

Thus, computing *P*(***t***_*D*+1_) = *P*(*t*_1_,…,*t*_*D*_,*t*_*D*+1_) is necessary. As *t*_1_,…,*t*_*D*_,*t*_*D*+1_ are jointly Gaussian, ***t***_*D*+1_ is Gaussian as well with distribution [16]:

$$P(t_{d+1})=N(0,[\begin{array}{cc} K+\sigma_{d}^{2}I & k\\ k^{T} & k(x_{d+1},x_{d+1}) \end{array}])$$ (11)

where the elements of **k** are given by k_(*i*)_ = *k*(***x***_*i*_,***x***_*D*+1_) for *i* = {1,…,*D*}. Then, the conditional distribution in Eq (10) is the conditional distribution of two Gaussian functions. The mean and variance of Eq (10) are then [16]:

$$\mu_{t_{D+1}|t}=k^{T}{\left(K+\sigma_{d}^{2}I\right)}^{-1}t$$ (12)

$$\Sigma_{t_{D+1}|t}=k(x_{D+1},x_{D+1})-k^{T}{\left(K+\sigma_{d}^{2}I\right)}^{-1}k$$ (13)

Here, Eqs (12) and (13) are used to predict the mean and variance of *t*_*D*+1_ given input ***x***_*D*+1_,kernel function *k*, and training data. For predicting a series of new points t* with features x*, the scalar kernel function *k* is replaced with the covariance matrix of t*, and **k** becomes the *D*×D* covariance matrix between ***x*** and x*, where D* is the number of points in t*.

If the observations are known to have non-zero mean, the simple transformation of ***t***′ = ***t***−E[***t***] creates a new random variable ***t***′ with mean zero, and the GP estimator derivation proceeds identically, ultimately yielding:

$$\mu_{t^{*}|t}=\mu_{t^{*}}+k^{T}{\left(K+\sigma_{d}^{2}I\right)}^{-1}(t-\mu_{t})$$ (14)

The covariance is the same as in Eq (13).

#### Kernel functions overview

Kernel functions define the covariance between *x* and *x*′ in a feature mapped space without needing to explicitly define the feature transformation *ϕ*(*x*) [16]. As a note, kernel functions can have vector or scalar input. The notation in this section uses scalar inputs as it reflects the data at hand. Eq (6) provides the definition of a general kernel function. Perhaps the simplest kernel is the linear kernel, in which *ϕ*(*x*) =*x* such that the kernel function is:

$$k(x,x^{\prime })=xx\prime$$ (15)

**Fig 1:**
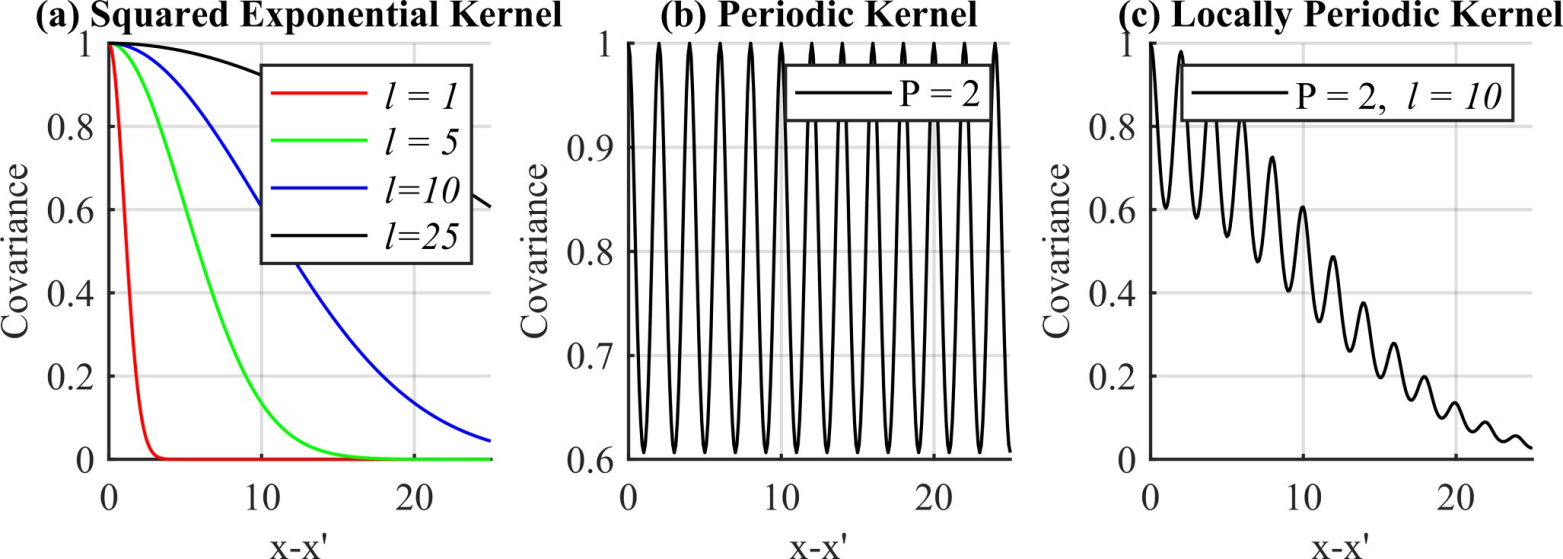
Kernel functions. Examples of the Squared Exponential (a), periodic kernel (b), and locally periodic kernel (c).

Not all functions are valid kernel functions, however. The primary condition to satisfy is that the Gram Matrix constructed from a given kernel must be positive semidefinite for all possible choices of *x* [16]. Since the purpose of a kernel-based approach is to conveniently define covariance, one can simply select a kernel which is known to be valid and captures the covariance in a desired way. Popular kernels include the Gaussian (or squared exponential) kernel (Fig 1A) [17]:

$$k_{G}(x,x^{\prime })=\sigma^{2}\;\exp\left(-\frac{{\left(x-x^{\prime }\right)}^{2}}{l^{2}}\right)$$ (16)

where l is the characteristic length which controls how quickly covariance decays, and *σ*^2^ is the output variance, analogous to the weight variance in Eq (6); and the periodic kernel (Fig 1B) [17]:

$$k_{P}(x,x^{\prime })=\sigma^{2}\;\exp\left(-\frac{2\;\sin^{2}\left(\frac{\pi |x-x^{\prime }|}{p}\right)}{l^{2}}\right)$$ (17)

where *p* defines the periodicity of the covariance, and *σ* and l are the same as in Eq (16). Here, l, *σ*, and *p* are examples of hyperparameters which need to be selected or optimized for over a given data set.

Valid kernels can also be combined or transformed to form new kernels to capture unique features. Given valid kernels *k*_1_(*x*,*x*′) and *k*_2_(*x*,*x*′), these can be transformed into a new valid kernel *k*_3_(*x*,*x*′) following a selection of permitted transformations and combinations such as:

$$k_{3}(x,x^{\prime })=c\;k_{1}(x,x^{\prime })$$ (18)

$$k_{3}(x,x^{\prime })=k_{1}(x,x^{\prime })+k_{2}(x,x^{\prime })$$ (19)

$$k_{3}(x,x^{\prime })=k_{1}(x,x^{\prime })\times k_{2}(x,x^{\prime })$$ (20)

Given Eq (20), the periodic and Gaussian kernels can be multiplied to form the locally periodic kernel (Fig 1C), where *r* = *x*−*x*′:

$$k_{LP}(r)=\sigma^{2}\;\exp\left(-\frac{1}{l^{2}}(d^{2}+2\;\sin^{2}\left(\frac{\pi |r|}{p}\right))\right)$$ (21)

Selection of a kernel function and its hyperparameters depends upon the data and is ultimately what determines the effectiveness and generalizability of the KBGP used. While methods for selecting kernels in some machine learning methods such as Support Vector Machines (SVMs) exist [18], the predominant method for choosing kernels in GP regression is by selecting a finite number of kernels which may provide good results given your data, testing each, and selecting from among them [19]. This inference approach requires the analyst know something about the data since an infinite number of kernels exits.

The primary distinction in classes of kernel functions are stationary vs. nonstationary [16]. Stationary processes satisfy *k*(*x*,*x*′) = *k*(*x*−*x*′) whereas nonstationary do not. This implies stationary processes are independent of shifts in input and rather depend solely on the distance between points, whereas nonstationary processes vary under these conditions. The aforementioned Gaussian kernel is a stationary kernel whereas the linear kernel is nonstationary. Smoothness, how well a kernel handles discontinuity, is another distinction in class. Stationary kernels should be selected for stationary processes, and smooth kernels should be selected for smooth data.

In the problem at hand, sparse spectrum data varies abruptly and discretely between neighboring channels, making it nonsmooth; but the overall distribution of source presence per channel varies relatively smoothly. In both cases, the process is stationary as distance between the channels dictates how much information we expect them to share.

#### Hyperparameter overview and selection

Kernel functions typically contain some number of hyperparameters which need to be selected prior to use. These include the previously mentioned characteristic length l and periodicity *p*. Hyperparameter selection can be as influential as the kernel itself as seen in Fig 1A. Typically, KBGPs go through a training phase where the hyperparameters are selected to optimize the fit over the training data. This is done by optimizing *P*(***t***|***θ***), where ***θ*** is the hyperparameter vector, for an optimal hyperparameter selection $\hat{\theta }$. While there are optimization strategies for computing $\hat{\theta }$, we omit these in this paper in favor of fixed, system-defined hyperparameters. This choice is motivated by the context of the problem and the time constraints it enforces: by eliminating the training for $\hat{\theta }$, the estimation time is kept to on the order of the dwell time.

#### Linear innovation sequences overview

The source estimation task has a sequence of inputs {***y***^(1)^,…,***y***^(*T*)^}, where $y^{\left(t\right)}=\{y_{1}^{\left(t\right)},\ldots ,y_{D}^{\left(t\right)}\}$, $y_{d}^{\left(t\right)}$ is the estimated source counts in channel *d* for the *t*^*th*^ collection, *T* is the number of collections, and *D* is the number of detector channels. The goal is to estimate the source presence ***z*** = {*z*_1_,…,*z*_*D*_} in each channel after the *T*^*th*^ newest collection given by $s^{\left(T\right)}=\left\{s_{1}^{\left(T\right)},\ldots ,s_{D}^{\left(T\right)}\right\}=E[z|y^{\left(1\right)},\ldots ,y^{\left(T\right)}]$. This task then has a training set of size (T*D) and testing set of size *D*. The covariance matrix **K** in Eq (14) is then of size (T*D)× (T*D). For example, for a detector with 1024 channels, by the tenth collection, the covariance matrix will be of size 10240×10240.

The size of the training data is problematic since the primary complexity cost of KBGPs is inverting the covariance matrix present in Eq (14). While methods for inverting large matrices quickly exist, such as Cholesky Decomposition [20], taking more than a couple seconds to do so breaks the criteria for the quick search method desired. An alternative to inverting an ever-increasing covariance matrix is needed. These computational scaling issues inherent to simple GP implementation have been alleviated via sparse GP approximations [21, 22, 23], but such approaches introduce additional complexities including the need to initialize large sparse matrices or access to utilize batches of training samples. These drawbacks are not of consequence under normal computational conditions, but here data enters the algorithmic pipeline serially in time and has no bound on survey period. Additionally, all computations will be done with on-board hardware (such as a smartphone), greatly limiting computational power. Finally, since we fully omit hyperparameter training, the benefits these advanced approaches offer are reduced.

Linear Innovative Sequences (LIS) provide a solution to this inversion problem, requiring the inversion of only the most recently acquired sequence to update an existing estimate. Namely, let ***z***,***y***^(1)^,…,***y***^(*T*)^ be random vectors with finite second moments. First, Linear Minimum Mean Squared Error (LMMSE) estimation provides a solution for the estimation of E[***z***|***y***^(*t*)^] as [24]:

$$\hat{E}[z|y^{\left(t\right)}]=E[z]+\mathrm{Cov}(z,y^{\left(t\right)})\mathrm{Cov}{\left(y^{\left(t\right)}\right)}^{-1}y^{\left(t\right)}$$ (22)

for ***y***^(*t*)^ with zero mean. For nonzero mean ***y***^(*t*)^, one can substitute these with ***y***^(*t*)^′ = ***y***^(*t*)^−E[***y***^(*t*)^]. Note the similarity in form between Eqs (22) and (14). In fact, if ***z*** is taken as test points t* and ***y***^(*t*)^ as training points ***t***, then these estimators are identical except for their covariance definition. Explicitly, Eq (14) relies upon a kernel definition while Eq (22) uses the traditional covariance definition. Second, for ***y***^(*t*)^ which satisfies *E*[***y***^(*t*)^] = 0 and $E[y^{\left(i\right)}{y^{\left(j\right)}}^{T}]=0$ for *i*≠*j* (orthogonality), it can be shown that [24]:

$$s^{\left(T\right)}=\hat{E}[z|y^{\left(1\right)},\ldots ,y^{\left(T\right)}]=E[z]+\sum_{t=1}^{T}\hat{E}[z-E[z]y^{\left(t\right)}]$$ (23)

This provides the solution to our desired estimation. However, we do not know E[***z***] by definition of our problem. Neither can we say that *E*[***y***^(*t*)^] = 0 or are orthogonal outright. LIS provide a direct solution for imposing *E*[***y***^(*t*)^] = 0 and orthogonality via the transformation [24]:

$${\tilde{y}}^{\left(t\right)}=y^{\left(t\right)}-\hat{E}[y^{\left(t\right)}|Y^{\left(t-1\right)}]$$ (24)

where ***Y***^(*t*−1)^ = {***y***^(1)^,…,***y***^(*t*−1)^} and:

$$\hat{E}[y^{\left(t\right)}|Y^{\left(t-1\right)}]=E[y^{\left(t\right)}]+\sum_{i=1}^{t-1}Cov(y^{\left(t\right)},{\tilde{y}}^{\left(i\right)})Cov{\left({\tilde{y}}^{\left(i\right)}\right)}^{-1}{\tilde{y}}^{\left(i\right)}$$ (25)

such that [24]:

$${\tilde{y}}^{\left(t\right)}=y^{\left(t\right)}-E[y^{\left(t\right)}]-\sum_{i=1}^{t-1}Cov(y^{\left(t\right)},{\tilde{y}}^{\left(i\right)})Cov{\left({\tilde{y}}^{\left(i\right)}\right)}^{-1}{\tilde{y}}^{\left(i\right)}$$ (26)

and we define ${\tilde{y}}^{\left(1\right)}=y^{\left(1\right)}-E[y^{\left(1\right)}]$. Eq (26) defines the LIS. Substituting Eq (24) into Eq (23) yields the final form:

$$s^{\left(T\right)}=\hat{E}[z|Y^{\left(T\right)}]=\hat{E}[z|{\tilde{Y}}^{\left(T\right)}]=E[z]+\sum_{t=1}^{T}\hat{E}[z-E[z]|{\tilde{y}}^{\left(t\right)}]$$ (27)

Note, for each new sequence ***y***^(*t*)^ observed, updating the estimate only requires inverting $Cov({\tilde{y}}^{\left(t\right)})$ as all other inversions are computed and stored in past steps. Returning to the motivating example, instead of inverting a single (T*D)×(T*D) matrix on the *T*^*th*^ iteration, a *D*×*D* matrix is inverted instead. But, Eq (26) demands the covariance of ${\tilde{y}}^{\left(t\right)}$, where we require the inverse of $\left(K+\sigma_{d}^{2}I\right)$ for the KBGP previously described. These are fortunately the same thing because in the GP derivation, we imposed Cov(***y***) = **K** in Eq (5), where ***y*** is our observation, as it is here.

Addressing the final undefined term, it can be shown that $Cov(y^{\left(t\right)},{\tilde{y}}^{\left(i\right)})=0$ in Eq (26). This relies on the assumption that each collection is independent such that: Cov(***y***^*(*i)^,***y***^(*j*)^) = 0 for *i*≠*j*. This result simplifies the incorporation of LIS, with the final results being:

$$s^{\left(T\right)}=\hat{E}[z|Y^{\left(T\right)}]=E[z]+\sum_{t=1}^{T}Cov(z,{\tilde{y}}^{\left(t\right)})Cov{\left({\tilde{y}}^{\left(t\right)}\right)}^{-1}{\tilde{y}}^{\left(t\right)}$$ (28)

And in the GP notation established previously:

$$s^{\left(t\right)}=\mu_{z|Y}=\mu_{z}+\sum_{i=1}^{t}k^{T}{\left(K+\sigma_{d}^{2}I\right)}^{-1}{\tilde{y}}^{\left(i\right)}$$ (29)

where ***y***^(*t*)^ is the *t*^*th*^ estimated source spectrum. Finally, the undefined **μ**_z_ is the estimate from the previous collection step. For example, the first collection assumes **μ**_***z***_ = 0, as GPs do. The second collection will assert **μ**_**z**_ =***s***^(1)^ and so on. Thus, the mean of the source distribution is updated after each collection step.

### Algorithm overview

**Fig 2:**
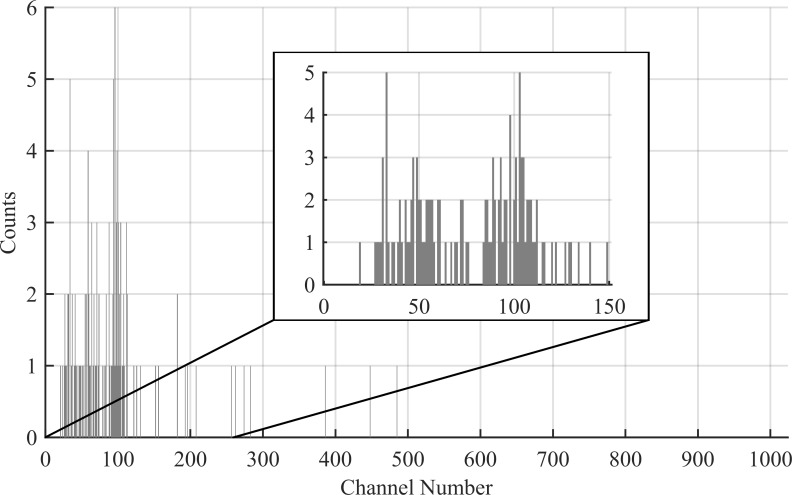
Example spectrum. Example of a sparse gamma ray spectrum with a weak source peak with centroid at channel 100.

**Fig 3:**
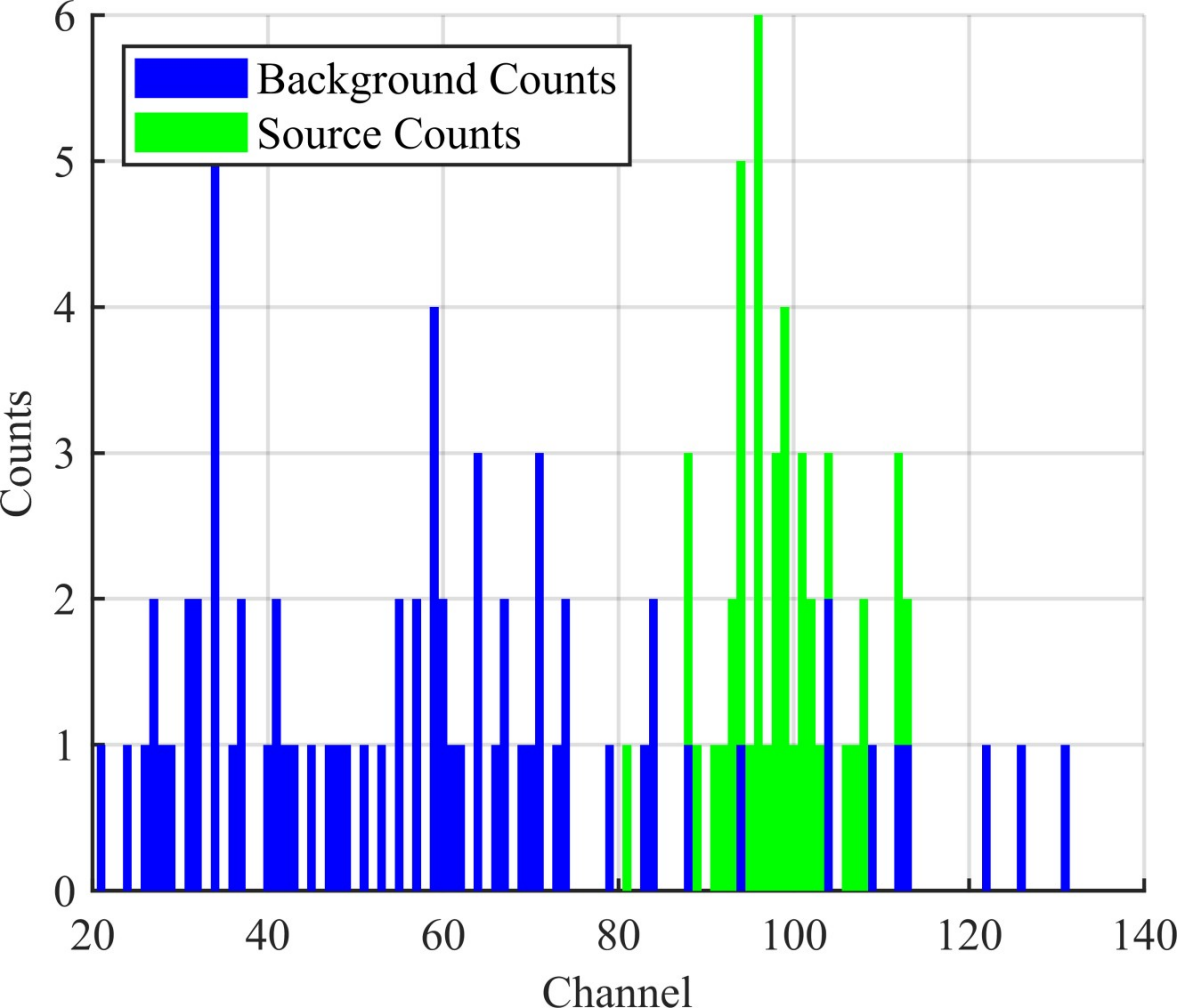
Labelled spectrum. Input spectrum from Fig 2 with explicitly labelled source counts. The algorithm does not see this labelled information.

The algorithm for anomaly detection is broken into two parts: the first part is a KBGP for noise reduction (KBGP-NR), and the second part is a KBGP for source distribution estimation (KBGP-SE). The KBGP-NR views only the most recent collection and reduces noise in the spectrum. The KBGP-SE considers all noise reduced collections and uses LIS to update an ongoing estimate of the source distribution. The estimated source distribution is analyzed for anomalies. The following sections provide an overview of these distinct algorithms. Fig 2 presents a typical sparse spectrum acquired with a D3S detector over 2 s and an injected source peak at channel 100. The training set generation is outlined in the validation portion of this report. Fig 3 presents the same spectrum with labelled source counts for comparison.

### KBGP-NR overview

The KBGP-NR is a GP used to reduce the presence of background in collected spectra leaving a greater source to background ratio for further analysis. The structure of the KBGP-NR is based upon that presented in [11], and so only the sparse data improvements and a brief overview are presented here.

Using the KGPB framework established previously, the input to this KBGP, *x*, is channel number and the output, *t*, is the number of counts attributed to background in channel *x*. The KBGP-NR is broken into three stages: 1) defining training set and testing sets from the most recent collection, 2) KBGP training and prediction, and 3) spectrum reconstruction.

#### 1) Training and testing sets

Stage one, called “spectral decomposition” in [11], involves decomposing a single collection into a training set representing background channels and testing set representing channels which have a mix of background and source counts. The training set, comprised of {channel number, counts} pairs, must adequately represent background and is thus defined by the spectrum minima. All counts in the minima channels are assumed to be purely background counts [11, 25, 26]. The remaining {channel number, counts} pairs form the test set and contain a mix of background and source counts; the goal is to estimate how many counts belong to background. For extremely sparse data, however, this definition is insufficient since the majority of channels are zero-count (minima) and many of the non-zero count channels are adjacent to these. This leads to the common situation where channels with any non-zero count are labelled as a nonminimum.

To resolve this issue, two spectra are collected (1 second of data each) rather than one. The minimum check is performed on each 1 second of data independently. If a given channel *d* is labelled as a minimum in either of the spectra, then the associated channel in their summed 2 s spectrum is labeled as a minimum (only background). A channel with no source presence should not consistently contain counts on such a short time scale, so using this technique to select against low count channels aids in defining a reliable training set.

#### 2) KBGP estimation

Now with a testing and training set, estimation commences. As mentioned previously, we omit training the hyperparameter training phase common for GP regression opting for a direct definition in order to reduce the time of estimation.

While the Matérn and Gaussian kernels tested in [11] were found to perform satisfactorily for their data, the samples considered here contain one tenth the counts. The data is extremely non-smooth and thus the semi-smooth Gaussian and Matérn kernels underperform. The locally-periodic kernel in Eq (21) is instead used as it better captures the discontinuous nature of the data over short length scales. The hyperparameters l and *p* are fixed at implementation. The parameter l is set to the full width at half maximum (FWHM) value at the peak resolution of the detector. The justification of this selection is that the detector cannot resolve below this threshold, and so the tails of any photopeak will just barely fall within this characteristic length. The periodicity *p* is set equal to 2 such that the discrete changes in counts between neighboring channels can be accounted for. Setting *p* = 2 (as seen in Fig 1C) increases the covariance between every second channel while selecting against immediate neighbors. The hyperparameter $\sigma_{d}^{2}$ is determined by computing the maximum variance in counts achieved over the characteristic length.

With a tuned kernel, the training set, and testing set, Eq (12) yields the estimated mean number of background counts in nonminima channels while Eq (13) provides the variance for each of those estimations.

#### 3) Source and background decomposition

The final stage decomposes the full spectrum into estimated background and estimated source spectra using the estimated mean background counts in mixed channels. Each tested channel (nonminima) undergoes the variance check and discretization from [11]. The variance check uses the variance computed from Eq (13) to test whether the recorded counts fall within two standard deviations of the mean background estimate; if so, then all counts in that channel are assigned to background, else only the estimated amount are and the remainder are assigned to source. Discretization is necessary as the output of the KBGP is not discrete but counts are.

Since the spectra are so sparse, however, an additional step was required to help offset the problem of many nearby minima (zero count channels in particular) causing the KBGP to place channels with few counts into the background spectrum despite having a high count neighbor channel. For any testing channel which had all counts placed into the background estimate after the variance check, if its immediate neighbors still contain source counts, then only the estimated counts are set as background instead of all.

**Fig 4:**
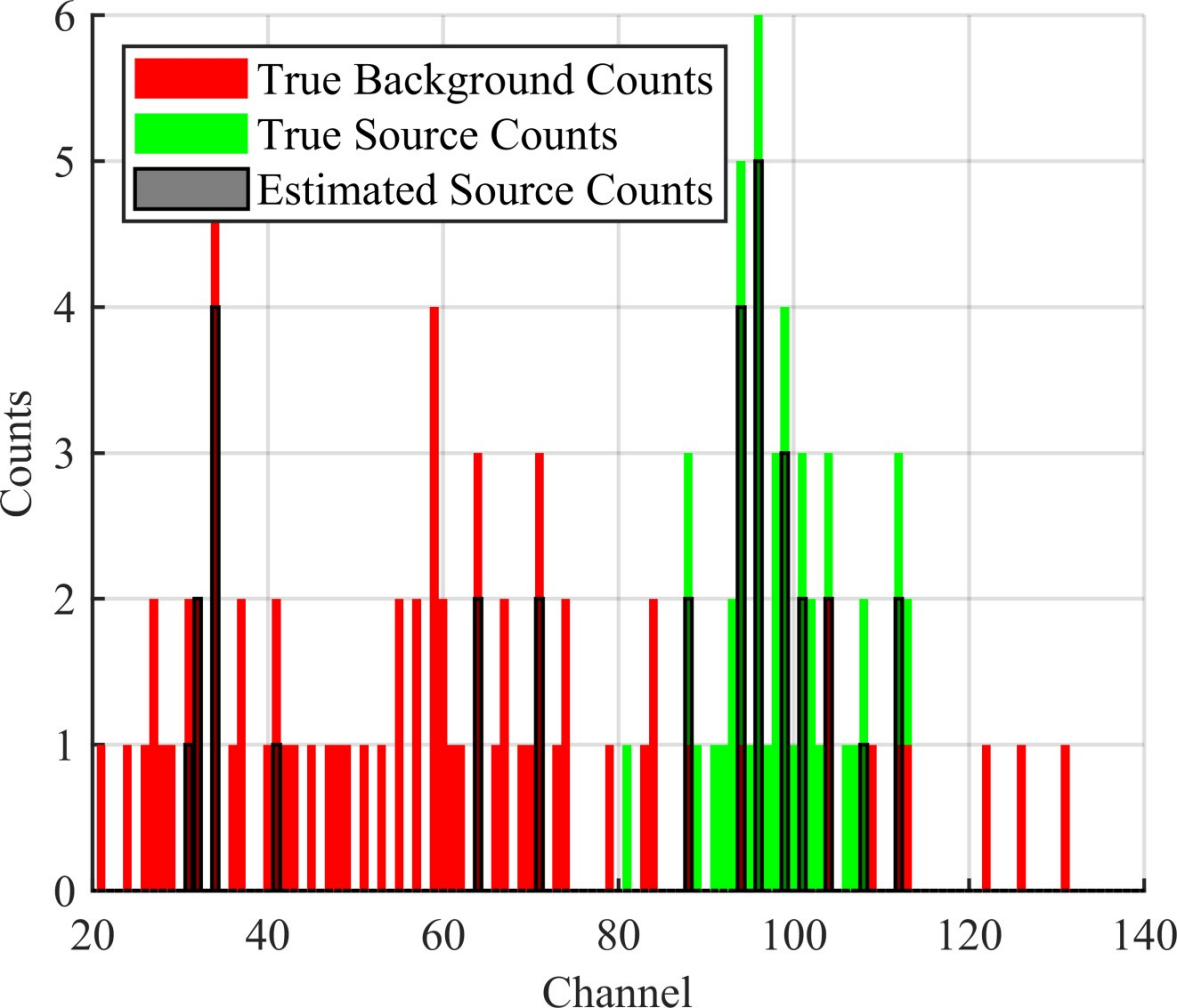
Labelled spectrum. Example spectrum with estimated source counts from the KBGP-NR labelled in blue. These estimated counts are the input into sure KBGP-SE.

Thus, the output consists of an estimated background spectrum and estimated source spectrum each of length *D*. This output can be seen in Fig 4.

### KBGP-SE overview

Relying on the noise reduced output from the KBGP-NR, the KBGP-SE is for the task of source distribution estimation and anomaly detection.

Each noise reduced collection ***y***^(*t*)^ can be viewed as a sampling from the true source distribution ***z***, and the KBGP-SE uses these samples to build a mean estimate for it. Each new noise reduced collection ***y***^(*T*+1)^ is added to a sequence of observations ***Y***^(***T*+1**)^ = {***y***^(1)^,…,***y***^(*T*)^,***y***^(*T*+1)^} and all channels ***x*** need be tested for source presence. Thus, the estimate for the true source distribution ***z*** is computed with Eq (28). Utilizing the LIS scheme for updates reduces the complexity of the problem as compared to an ever-growing observations vector. The estimation cycle is separated again into three stages: 1) prepare the training set with weighting, 2) KBGP prediction with LIS, and 3) source estimation and anomaly detection.

#### 1) Training set

In order to build a training set, stage one processes the noise reduced collections from the KBGP-NR by weighting each channel with the inverse of the number of neighboring zeros. Since higher count density is expected in photopeak regions, this process helps select against poorly filtered background peaks. This weighting scheme counts the number of zeros around channel *d* with a window of size l across all *T* collections; let this vector be noted as ***w*** where the *d*^*th*^ element is:

$$w_{d}=\sum_{t=1}^{T}\sum_{i=d-\frac{l}{2}}^{d+\frac{l}{2}}\delta (y^{\left(t\right)}(C_{i}))$$ (30)

The counts in each channel are then divided by the square of this zero-weight and normalized across each collection:

$$y_{d}^{\left(t\right)}\prime =\frac{y_{d}^{\left(t\right)}}{w_{d}^{2}}{\left(\sum_{i=1}^{D}\frac{y_{i}^{\left(t\right)}}{w_{i}^{2}}\right)}^{-1}$$ (31)

where *D* is the number of channels. Following the LIS requirement, our training set is then:

$${\tilde{y}}^{\left(t\right)}=y^{\left(t\right)}\prime -E[y^{\left(t\right)}\prime ]$$ (32)

where E[***y***^(*t*)^′] is directly computable as the average counts in each channel. This weight vector is updated after every collection.

#### 2) KBGP estimation with LIS

Stage two uses Eq (28) to compute the mean of the estimated source distribution. The Gaussian kernel function Eq (16) with characteristic length l set equal to the detector resolution was chosen for the source estimation regression task because the expected source distribution will be smooth. The summation in Eq (28) is over *T* collections and where μ_**z**_ = ***y***^(*T*−1)^ for *t*>1. The hyperparameter $\sigma_{d}^{2}$ is also defined at this point as:

$$\sigma_{d}^{2}=\sigma_{h}^{2}+\sigma_{spectrum}^{2}$$ (33)

where $\sigma_{h}^{2}$ is the variance in counts *C* in channel *d* across all collections *T*:

$$\sigma_{h}^{2}=\frac{1}{T}\sum_{t=1}^{T}{\left({y_{d}^{\left(t\right)}(C}_{d})-E[{y_{d}^{\left(t\right)}(C}_{d})]\right)}^{2}$$ (34)

And $\sigma_{spectrum}^{2}$ is the variance across all channel measurements:

$$\sigma_{spectrum}^{2}=\frac{1}{TD}\sum_{t=1}^{T}\sum_{d=1}^{D}{\left({y_{d}^{\left(t\right)}(C}_{d})-E[y(C)]\right)}^{2}$$ (35)

#### 3) Source estimation and anomaly detection

The final stage takes mean estimate after each new collection to build an updated source distribution estimate and performs anomaly detection. Since the output of the KBGP-SE ***s***^(*T*)^ can be negative, but counts cannot be, any channel containing negative values is set equal to zero. Negative estimates occur in channels predicting approximately zero but which are near channels with higher count rates. Then, since the training data was scaled down with weighting, the output spectrum ***s***^(*T*)^ is scaled by the cumulative counts in the noise reduced collections, $s_{NR}^{\left(t\right)}$:

$${\hat{s}}^{\left(T\right)}=(\frac{s^{\left(T\right)}}{\sum_{d=1}^{D}s_{d}^{\left(T\right)}})\sum_{d=1}^{D}\sum_{t=1}^{T}{s_{NR}^{\left(t\right)}}_{d}$$ (36)

**Fig 5:**
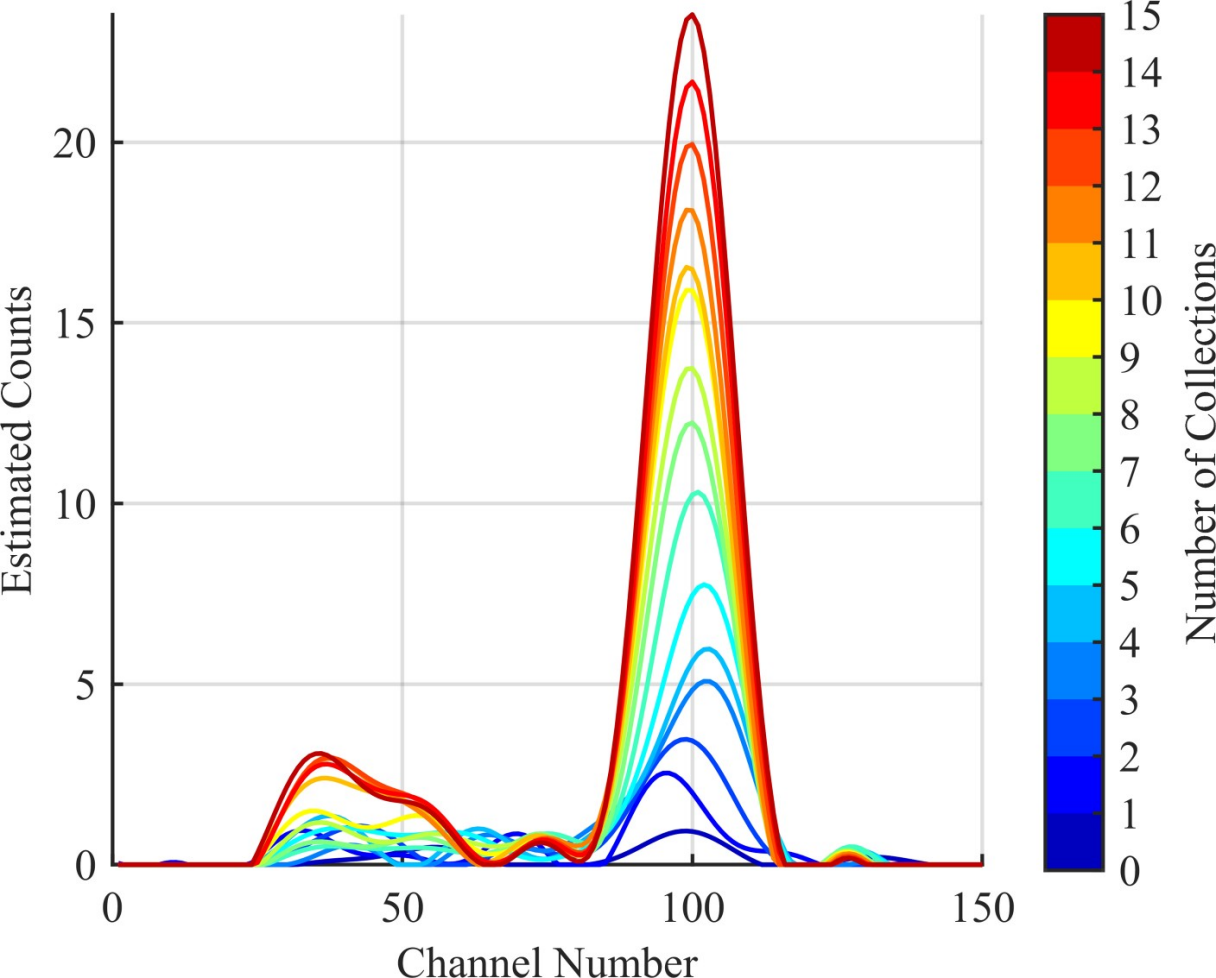
Updating source estimate. Series of the estimated source distribution after each of 10 collections. Note the source peak (channel 100) raises at a much faster rate than the noise peaks (all other peaks).

**Fig 6:**
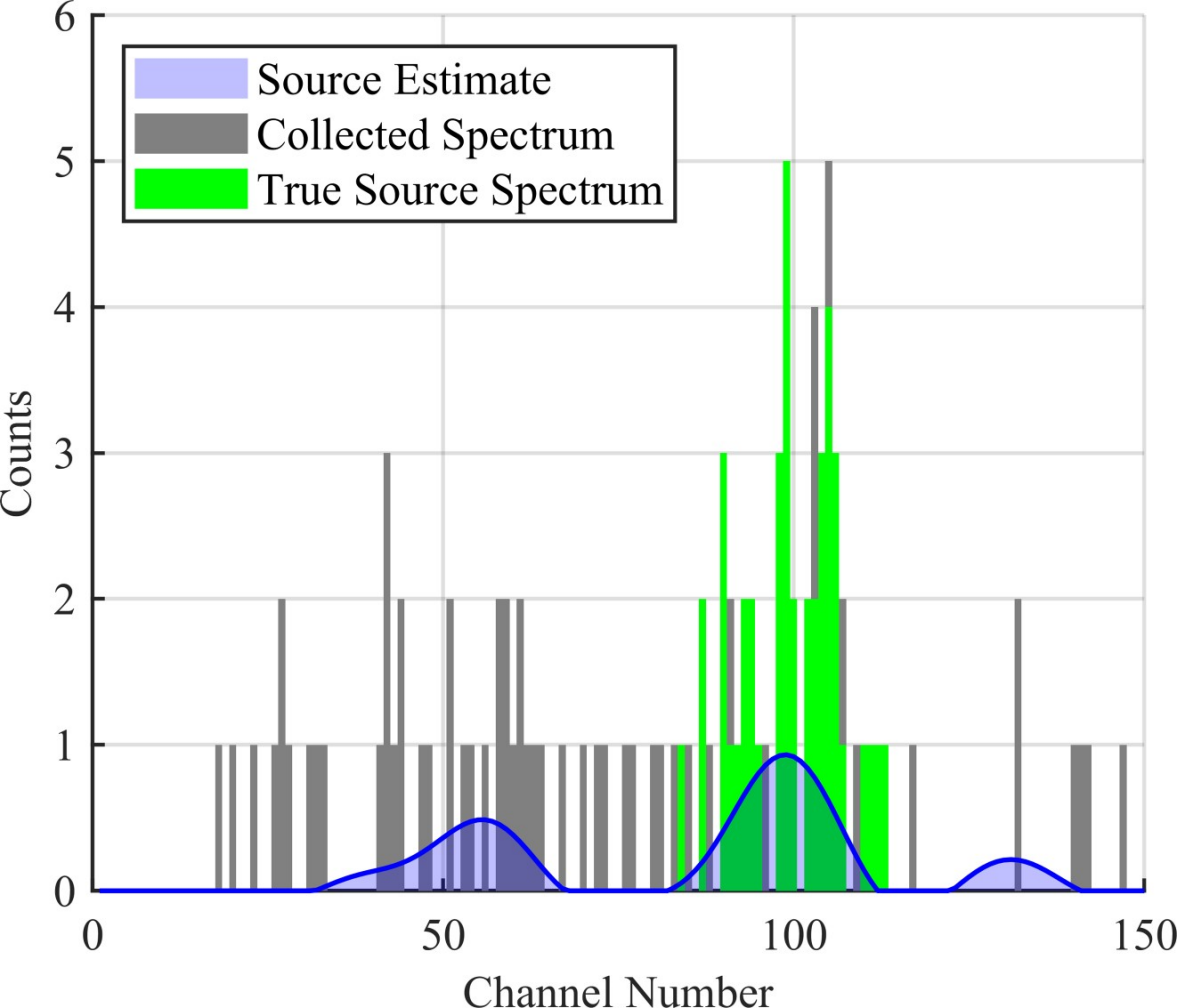
Source estimate after 1 collection. The estimated source distribution is given in blue, the collected spectrum is given in black, and the true source spectrum is given in green. The collected spectrum is 2 s worth of data.

**Fig 7:**
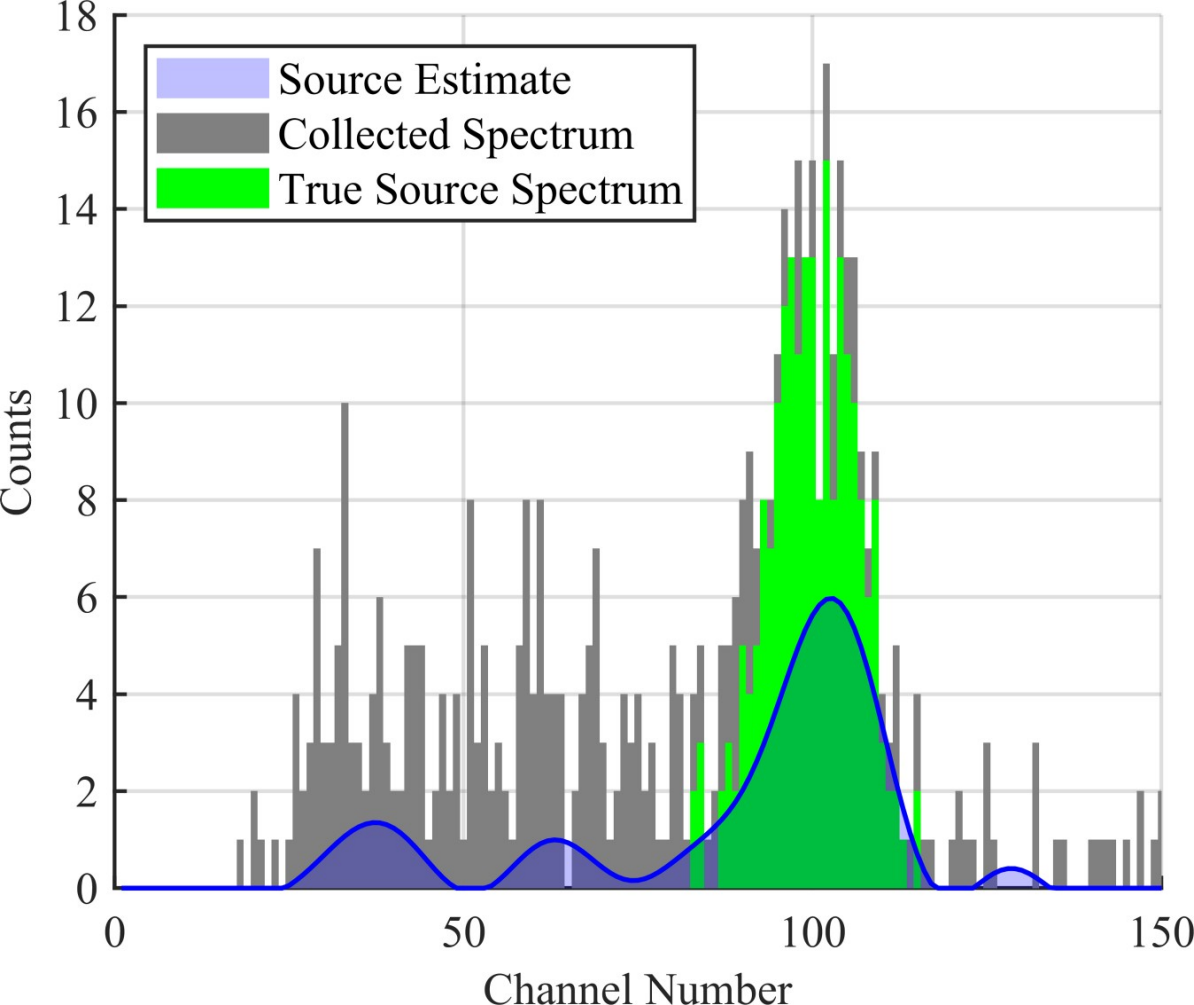
Source estimate after 5 collections. The estimated source distribution is given in blue, the cumulative collected spectrum is given in black, and the true source spectrum is given in green. The collected spectrum is 10 s worth of data.

**Fig 8:**
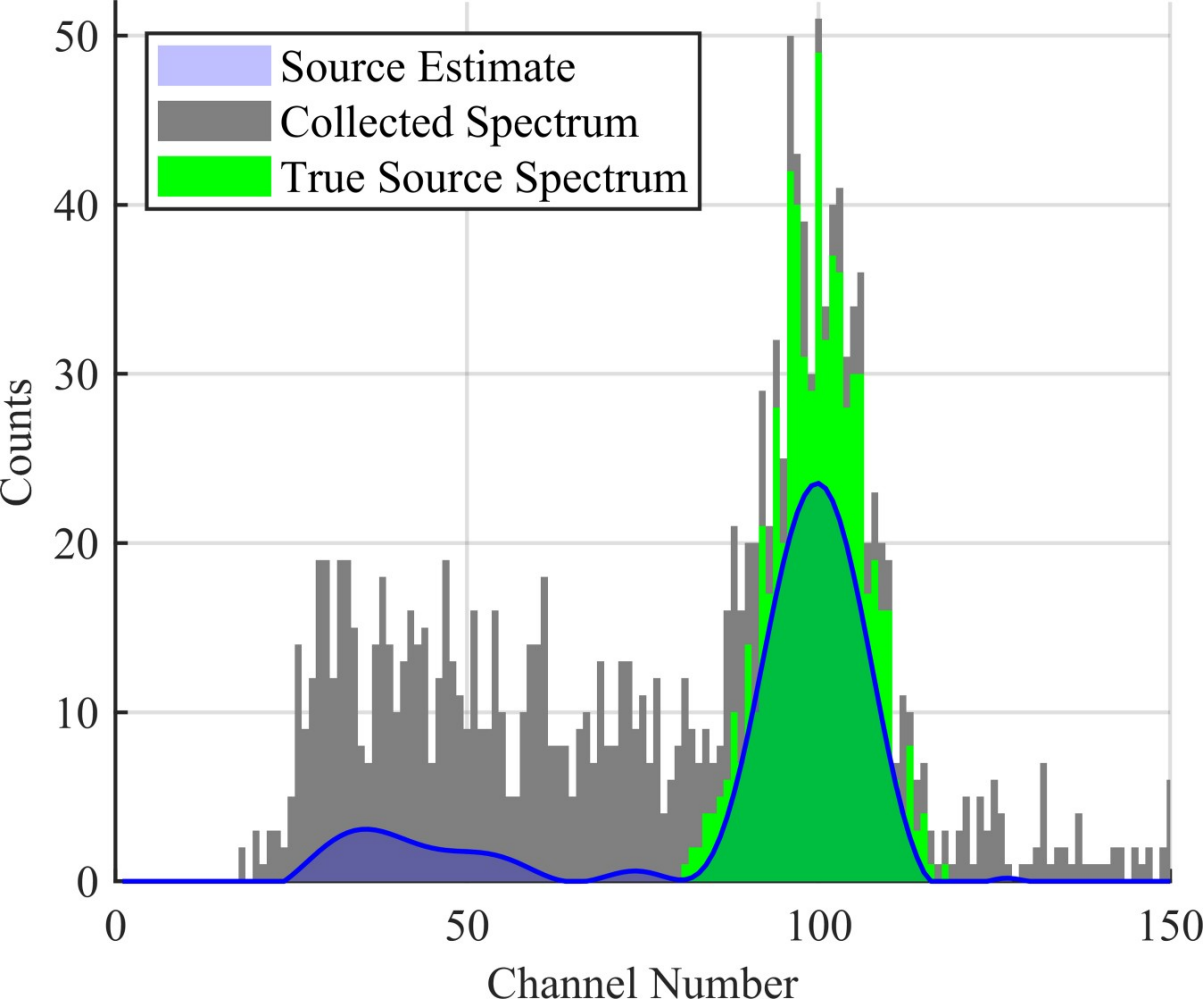
Source estimate after 15 collections. The estimated source distribution is given in blue, the cumulative collected spectrum is given in black, and the true source spectrum is given in green. Note the estimated noise peak as compared to the actual counts in corresponding channels. The collected spectrum is 30 s worth of data.

Here, ${\hat{s}}^{\left(T\right)}$ updates after each collection and provides a smooth estimate for the source distribution over all detector channels. This can be seen in Fig 5 over the course of 10 collections. This estimated distribution can be seen plotted against the cumulative collected spectrum in Figs 6, 7 and 8 corresponding to collection numbers 1, 5, and 15, respectively.

The features of $\hat{s}$ are used to determine location of anomalies by acquiring the height (counts) and location (channel) of maximums. Each maximum is then tested against a Gaussian peak as the KBGP-SE produces Gaussian features due to choice of kernel function. An area [*d*_*lower*_,*d*_*upper*_] is drawn around each maximum where: *d*_*lower*_ is the first point left of the maximum where the value is equal to or less than one percent of the peak’s height, reaches zero, or begins to rise again; and *d*_*upper*_ is the first point right of the maximum which meets the same criteria. A Gaussian distribution is then fit to the cumulative output of the KBGP-NR source estimate over this range:

$$s_{NR}^{\left(1:T\right)}=\sum_{t=1}^{T}{s_{NR}}_{\left[d_{lower},d_{upper}\right]}^{\left(t\right)}$$ (37)

If the *R*^2^ value computed for this fit is less than a defined threshold, then the peak is ignored for this collection. This is referred to as the *R*^2^ test. Poor fits are caused by either a small number of counts or peaks formed from background counts which leaked through from the KBGP-NR. Peaks formed from leaked background are not Gaussian and so fit poorly to a Gaussian distribution. If the *R*^2^ value is greater than a determined threshold, then this peak is considered subject to a density test. If two-thirds of the area under the curve of $\hat{s}$ is within [*d*_*lower*_,*d*_*upper*_], then the peak is considered an anomaly. The relative area under the curve over a specific window is referred to as “density”.

## Results

This section summarizes the testing of the KBGP-NR and KBGP-SE portions of the algorithm, keeping in mind the latter depends upon the output of the former. Firstly, the source and background spectrum acquisition protocol is summarized; then the KBGP-NR and KBGP-SE are tested separately.

### Source and background spectrum

The background test data was collected with a Kromek D3S gamma-ray detector on the University of Illinois at Urbana-Champaign campus with no sources other than NORM present. This background set consists of 14077 sample spectra, each 1 s collections, with an average count rate of 40±16 counts/s. An artificial source spectrum was synthesized via a statistical model. With a given source activity *a* and dwell time *t*, for each spectra, a Poisson random number was computed representing the total emissions *λ* as *λ*~*Pois*(*at*). With a defined distance *d* meters from the source and the face dimensions of the detector (0.5 in × 2.54 in), the geometric efficiency *ϵ*_*g*_ was calculated and used to compute the number of counts captured by the detector as *c* = *ϵ*_*g*_*λ*. The standard deviation of the detected count spread *σ* was computed using the FWHM of the detector. Detected counts *c* were then placed in the source spectrum around channel *d* according to a random Normal distribution following *N*(*d*,*σ*^2^). This peak is computed for each second of dwell time independently.

Since these tests are meant to be performed under sparse conditions, a source count rate of 20 counts/s, half that of the background count rate, was selected. This roughly equates to an activity of 2.2 mCi at a distance of 10 m. The source peak was placed at channel 100, just overlapping the noise region. For source peaks at higher channel numbers, the source counts are more easily distinguished from background. For source peaks at lower channel numbers, the background counts add to source peaks consistently, reducing the number of local minima, reinforcing the source peak location.

The source spectrum is then injected into the background spectrum such that a ground truth about background and source spectrum are known for each collection.

### KBGP-NR Validation

The validation of the noise removal algorithm consisted of a test with source present and another where no source is present. The metric of each test was how well the background and source spectra were separated as quantified by the correlation coefficient between the respective true and estimated spectra. Each test consisted of thirty independent trials using 2 s collections each. These results are compared against the results presented in [11] since these are functionally similar algorithms with ours being tuned for extremely sparse spectra. The algorithm from [11] was incorporated in full except for the hyperparameter tuning, opting instead for the fixed parameter approach.

For pure background, the proposed method provides an average correlation coefficient of 0.76±0.08 between the estimated background and true background spectrum over the thirty trials; the background estimation scheme from [11] yields a correlation coefficient of 0.36±0.14 between estimated background and true background. The discrepancy of the reported 0.816 correlation coefficient from [11] and the one present here comes down to the sparsity of the data used. Without implementing the additional strategies for selecting against noise, the sheer abundance of zero count channels leads channels with counts to be improperly placed in the source spectrum.

With a 2.2 mCi source placed 10 m from the detector, thirty 2 s source spectra were created. These were then injected into background spectra. The average correlation coefficient of estimated background to true background spectrum was 0.629±0.15; and the estimated source to true source spectrum average correlation coefficient was 0.67±12. Implementing the KBGP from [11] as before yields a background correlation coefficient of 0.33±13 and a source correlation coefficient of 0.60±0.08.

These tests indicate that the proposed method for background and source spectra prediction improves over [11] in the case of extremely sparse data and fixed hyperparameters. However, there is still sub-optimal estimation of source spectrum as indicated by the relatively low correlation coefficients. This motivates the need for the KBGP-SE.

### KBGP-SE Validation

The KBGP-SE tests consisted of, like in the previous section, one experiment with a source and another without. Each of these experiments consisted of 100 trials taking 15 successive collections of 2 s measurements each to make use of the updates available to the KBGP-SE. Each collection was first passed through the KBGP-NR to reduce the noise.

In observing the output from the KBGP-SE in Figs 6, 7 and 8, two notable peaks form: a peak centered near channel 100 correlating to source and another around channel 40 correlating to noise. This can be seen in Fig 7 and Fig 8. The noise peak is present with or without source presence. While some smaller peaks are identifiable, the results presented here focus on the noise peak which demonstrated the strongest chance to incorrectly pass the anomaly detection criteria.

**Fig 9:**
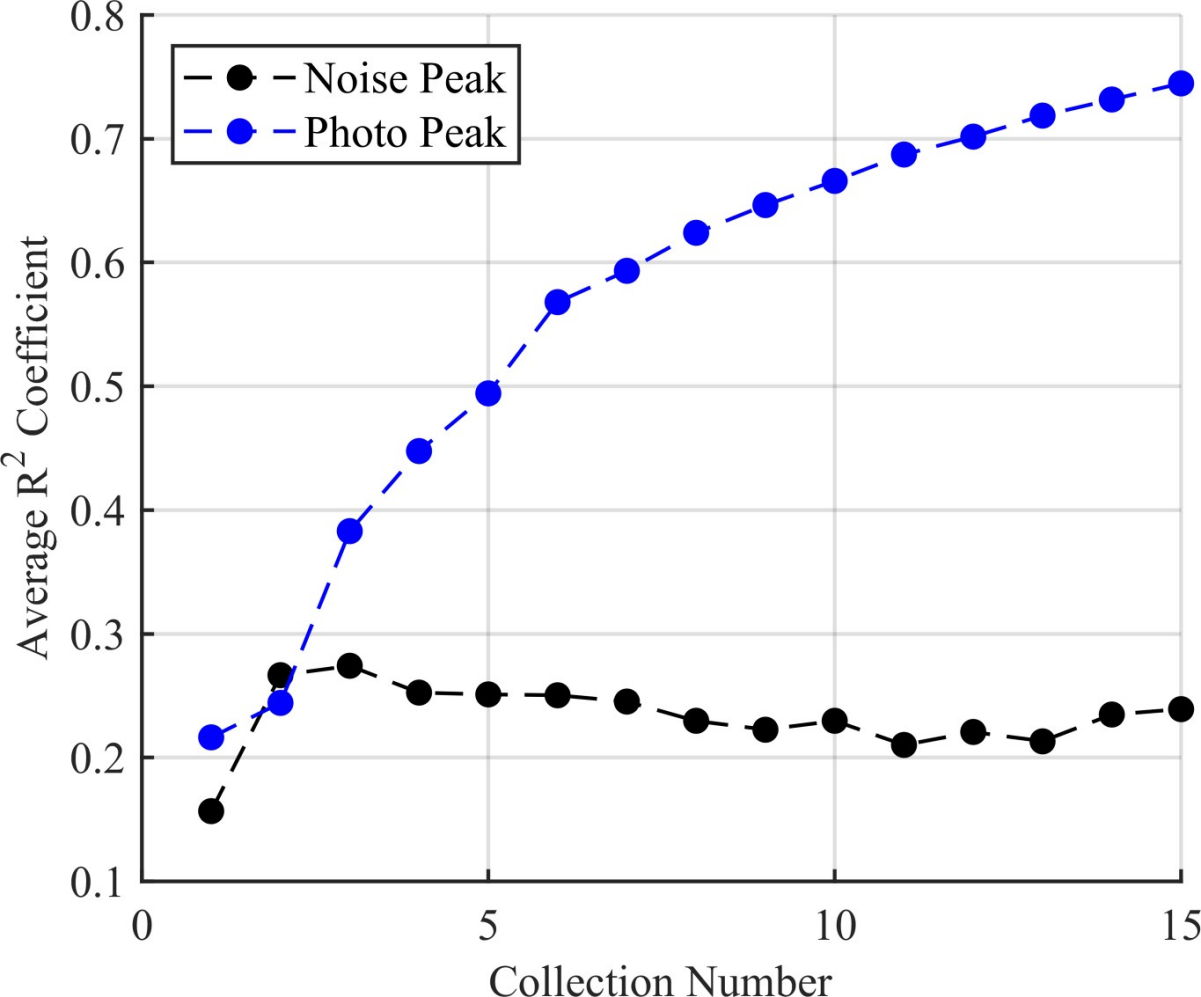
The average *R*^2^ test results for the 100 trials with source present. The source peak is increasingly Gaussian with each successive collection whereas the most convincing noise peak is poorly fitted over all collections. While the average *R*^2^ value for noise never passes 0.3, this is averaged over all 100 trials, and so some noise peaks do surpass this threshold.

**Fig 10:**
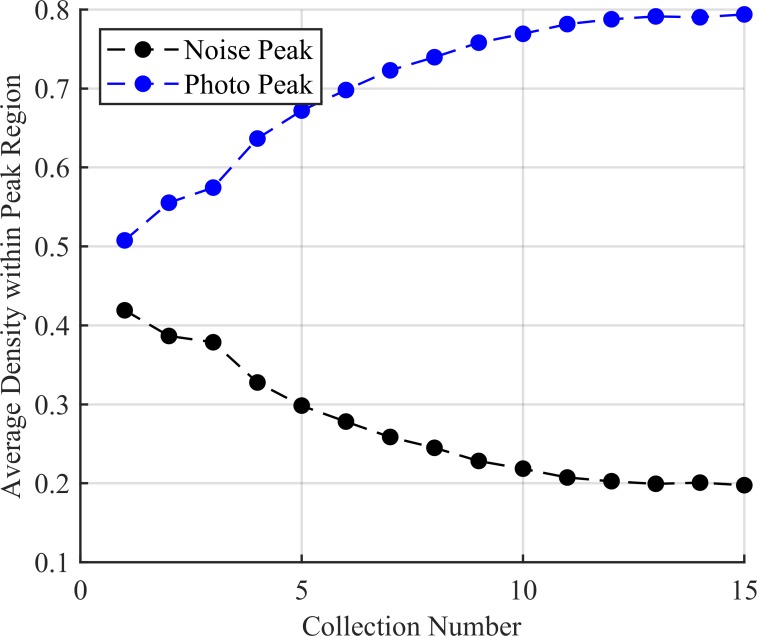
The average density of the noise and source peaks over the 100 trials with source present. The density within the source region increases with each successive collection while that of noise regions fall. This shows that the algorithm is correctly placing more counts in the source region with each new collection.

Both the noise and source peaks form Gaussian-like peaks due to the selection of kernel. The *R*^2^ coefficient used for the anomaly detection test rose with each successive trial for the source peak as the number of counts become sufficient enough to be represented as a Gaussian as seen in Fig 9. Here the most prominent noise peak *R*^2^ value only surpassed the photopeak *R*^2^ value in early collections. These were the average values over all 100 trials. It also demonstrates that the noise leaked through filtering becomes less Gaussian over time as the value falls over the successive collection. Relative density accumulated within the true peak region consistently while the noise region decreased in estimated source presence as seen in Fig 10.

**Fig 11:**
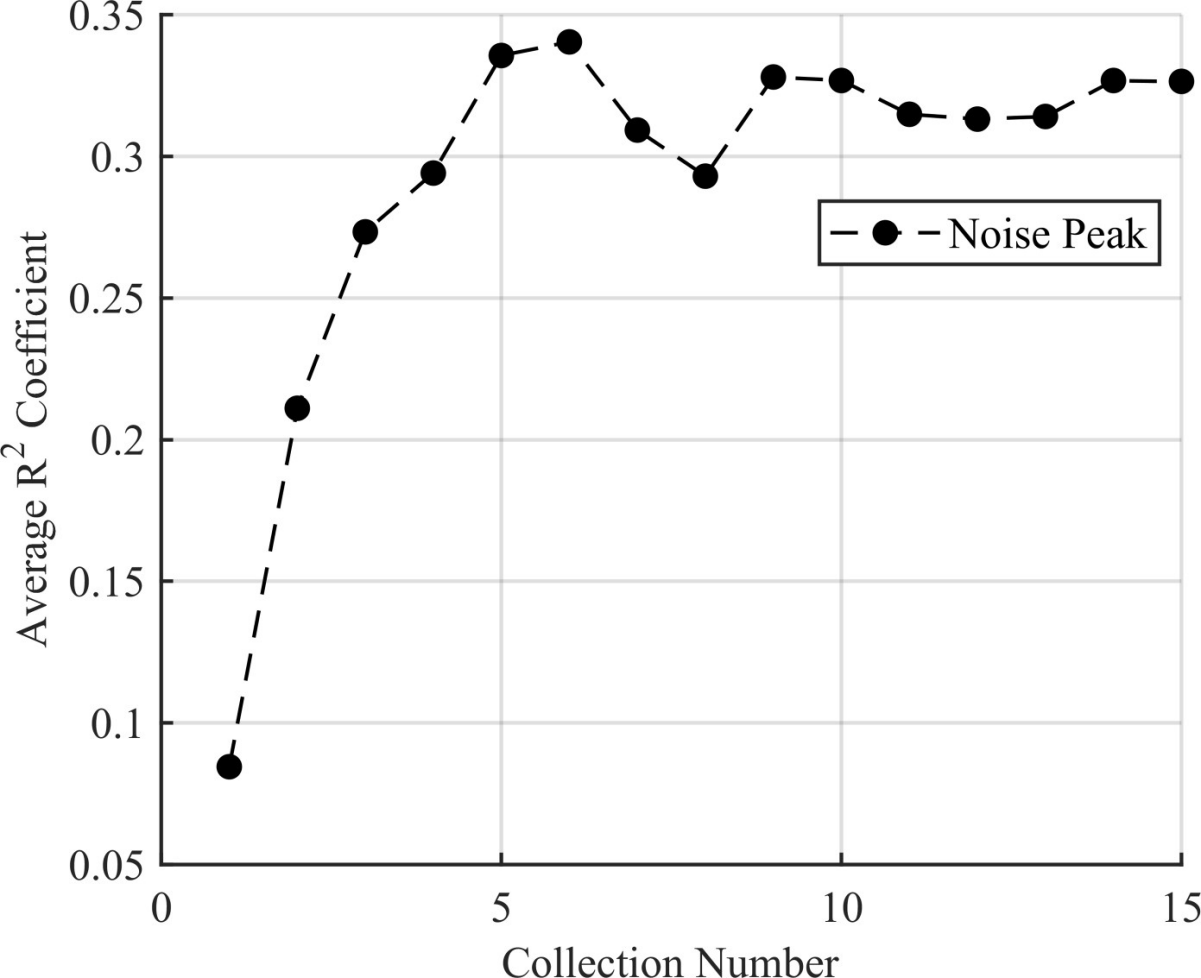
The average *R*^2^ test results for the 100 trials with no source present. The average *R*^2^ value for the best performing noise peak mirrors that of Fig 9, illustrating that the noise peak shape is independent of source presence.

**Fig 12:**
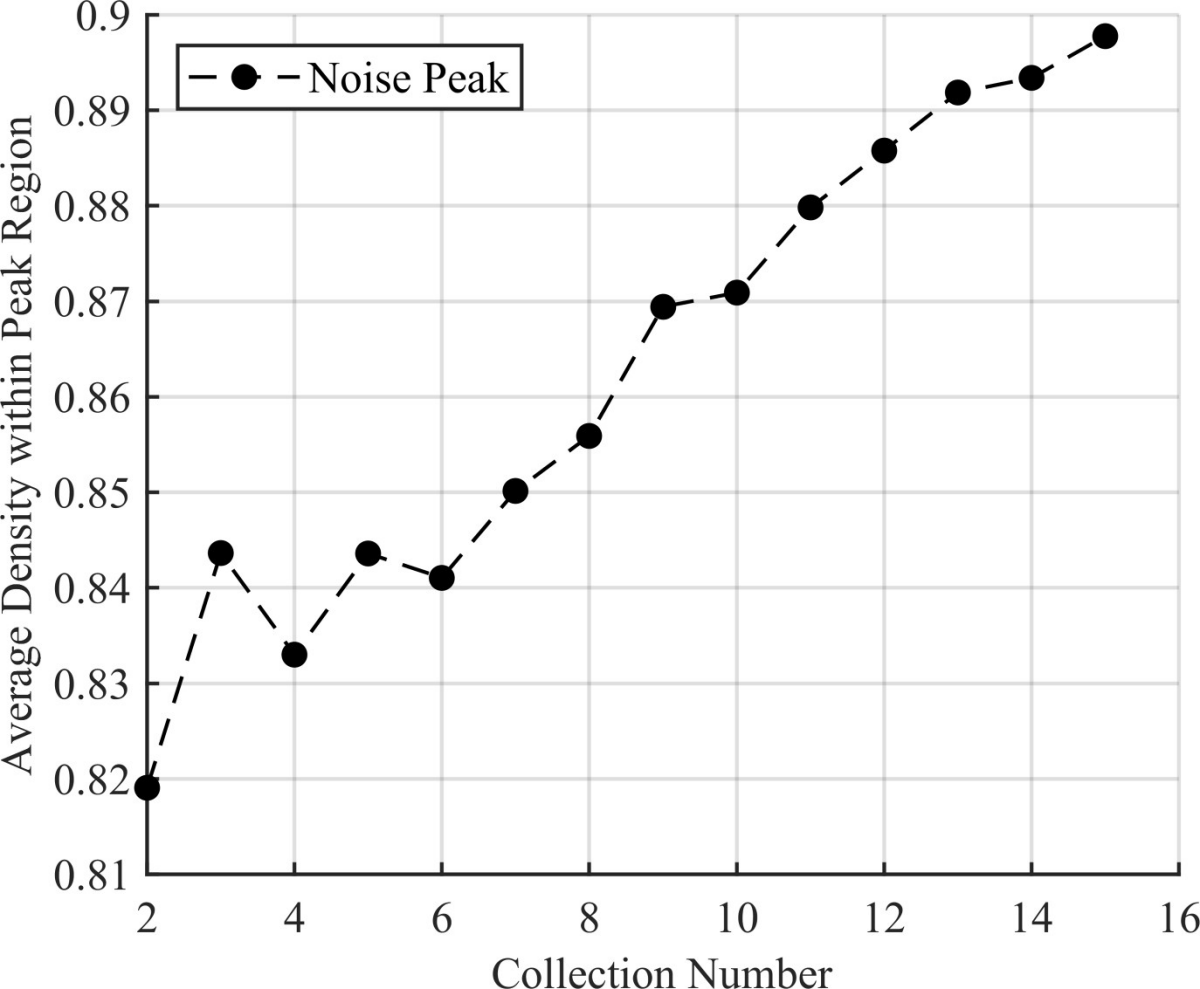
The average density of the noise peak over the 100 trials with no source present. While density is accumulating within the peak, this is done very slowly as indicated by the small percent increases with each new collection. This high value make sense as the noise region.

For the trials with no source, every trial passed the density test. This makes sense as only a single peak from leaked noise is present and so would necessarily contain the majority of the density. As such, the *R*^2^ test did a good job selecting again noise peaks as their distribution was not well captured by a Gaussian fit. The *R*^2^ value of the best scoring noise peak can be seen in Fig 11 and the density of the largest noise peak in Fig 12.

**Fig 13:**
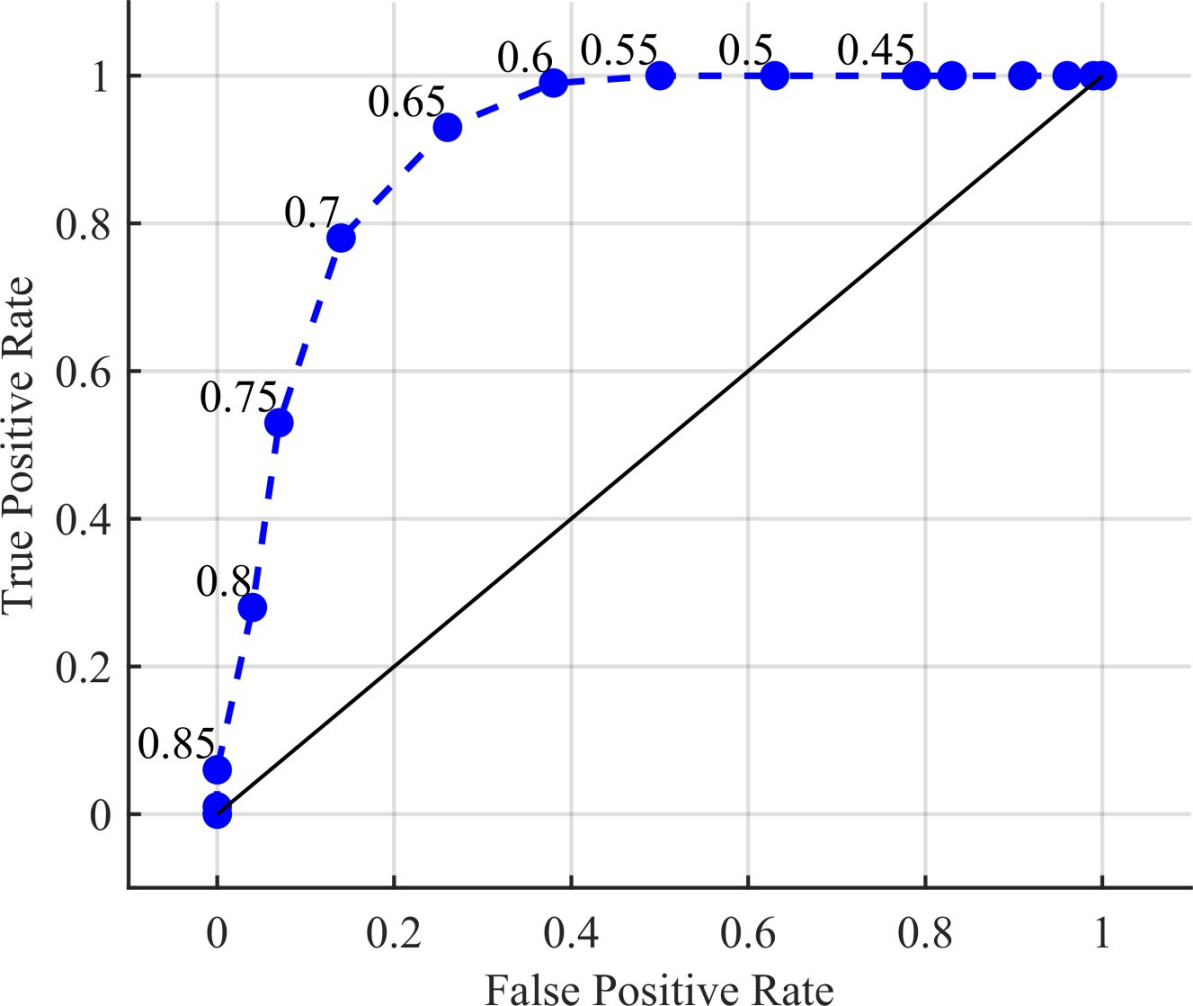
ROC curve for anomaly detection. False positive defined as claiming an anomaly is present when none are. True positive defined as claiming an anomaly is present when one is. The labels refer to the threshold set on the *R*^2^ test. If the *R*^2^ value is above this threshold, then the peak is considered anomalous.

The true positive rate for anomaly detection across the full algorithm (using the KBGP-NR for noise reduction and the KBGP-SE for source estimation) with an *R*^2^ threshold of 0.65 and averaged over all trials was 0.93. This can be improved upon by noting that these tests were conducted with a stationary detector. If paired with a surveying algorithm, as intended, the amount of information available to the algorithm should improve upon this accuracy. The ROC curve for anomaly detection while adjusting the *R*^2^ threshold can been seen in Fig 13. The area under the ROC curve was 0.902 demonstrating a good estimation process.

## Conclusion

Through a two-stage statistical model, we have developed an algorithm for anomaly detection for sparse data, source search scenarios. By using a KBGP for noise removal in order to have a more consistent source spectrum, we were able to demonstrate that by using successive sampling, a source peak can be identified in extremely sparse conditions and immune to selecting against noise peaks. While this algorithmic approach has been contextualized by a source surveying scenario, the detector position was held fixed in these trials, representing a scenario in which the surveyor never actually approaches the source. This equates to operating on only the worst quality information available during source surveying and still achieving the presented results.

While we demonstrated that this procedure is successful for a 2.2 mCi source 10 m from a low sensitivity detector whose photopeak just overlaps a noise dominated region, this algorithm can be applied to any number of combinations of source type, detector type, and distance. Most notably, this algorithm requires no training prior to use and is independent of gamma detector specifics except for a single hyperparameter governed by resolution. As such, it can be immediately incorporated into any sparse data gamma analysis for which a source location (peak, region, etc.) is required. Future work will explore using the source distribution estimate to bias against certain channels.

## Supporting information

S1 Table Background radiation measurements. The time column is in the unit of *epoch*. Each *s#* column represents the counts in channel *#*.
(CSV)
